# Effective weight control via an implanted self-powered vagus nerve stimulation device

**DOI:** 10.1038/s41467-018-07764-z

**Published:** 2018-12-17

**Authors:** Guang Yao, Lei Kang, Jun Li, Yin Long, Hao Wei, Carolina A. Ferreira, Justin J. Jeffery, Yuan Lin, Weibo Cai, Xudong Wang

**Affiliations:** 1 Department of Materials Science and Engineering, University of Wisconsin-Madison, Madison, WI 53706 USA; 2 State Key Laboratory of Electronic Thin films and Integrated Devices, University of Electronic Science and Technology of China, Chengdu, Sichuan 610054 People’s Republic of China; 3 Department of Radiology, University of Wisconsin-Madison, Madison, WI 53705 USA; 4 Department of Nuclear Medicine, Peking University First Hospital, Beijing, 100034 People’s Republic of China; 5 University of Wisconsin Carbone Cancer Center, Madison, WI 53705 USA

## Abstract

In vivo vagus nerve stimulation holds great promise in regulating food intake for obesity treatment. Here we present an implanted vagus nerve stimulation system that is battery-free and spontaneously responsive to stomach movement. The vagus nerve stimulation system comprises a flexible and biocompatible nanogenerator that is attached on the surface of stomach. It generates biphasic electric pulses in responsive to the peristalsis of stomach. The electric signals generated by this device can stimulate the vagal afferent fibers to reduce food intake and achieve weight control. This strategy is successfully demonstrated on rat models. Within 100 days, the average body weight is controlled at 350 g, 38% less than the control groups. This work correlates nerve stimulation with targeted organ functionality through a smart, self-responsive system, and demonstrated highly effective weight control. This work also provides a concept in therapeutic technology using artificial nerve signal generated from coordinated body activities.

## Abstract (web summary)

Developing new technologies for the neuromodulation of the vagus nerve can enable therapeutic strategies for body weight control in obese patients. Here, the authors present a battery-free self-powered implantable vagus nerve stimulation system that electrically responds to stomach movement.

## Introduction

Obesity resulted from ingesting calories in excess of normal biological requirement is a major risk for a large number of chronic diseases, including cardiovascular disease^1,2^, diabetes mellitus^1,3^, chronic kidney disease^1^, gallbladder diseases^4^, certain cancers^3,5^, musculoskeletal disorders^6^, and even genetic variation^7^. Treatment of obesity imposes an enormous economic burden on the global healthcare system^8–10^. According to a recent global survey, over 710 million people worldwide, including 107.7 million children and 603.7 million adults, are plagued by obesity problems, and about 4 million people died of overweight- or obesity-related diseases in 2015^10^. Common approaches for treating obesity include non-surgical and surgical treatments. Daily physical exercise and taking weight-loss drugs are common non-surgical weight reduction regimens, but there is a high potential of weight rebound or side effects from drugs^11^. Current bariatric surgical procedures such as gastric bypass, biliopancreatic diversion, and sleeve gastrectomy have demonstrated a significant impact on weight loss, but these procedures are invasive with the potential of serious complications^12–14^. The rising healthcare standards demand new obesity treatment strategies that are effective, easy to operate, and have less side effects.

Neuromodulation, as a non-destructive and reversible therapeutic strategy, can manipulate body functions by stimulating or influencing neurophysiological signals through the neural networks to achieve therapeutic purpose^15,16^. It has been known for a century that the vagus nerve (tenth cranial), a mixed parasympathetic nerve containing both afferent and efferent nerve fibers, acts as a signal bridge to transport information between the brain (the center of the nervous system) and the body (head, neck, thorax, and abdomen)^17–19^. Recent breakthroughs in neuromodulation for body weight control have provided potential opportunities for therapeutic interventions and brought renewed promises and vitality to the development of new anti-obesity strategies. A number of studies have demonstrated that pulsed electrical stimulations on vagus nerve could induce multiple physiologic functions related to food intake, energy metabolism, and glycemic control, which can result in appreciable weight loss^20–22^. An implantable vagus nerve stimulation (VNS) device for weight control was recently approved by Food and Drug Administration and commercialized^20,23^. Major concerns of current electrical stimulation are potential compensation mechanisms that blunt physiological responses^14^ and vicinity tissue damage that induces adverse effects^17,24,25^. In addition, the electrical system is bulky and complicated in operation. All the electrical stimulations need to be programed externally and the device needs to be charged periodically^13,26^. How to achieve real-time-responsive and self-sustainable stimulation remains a major challenge for this promising weight control strategy.

In this work, we present a correlated VNS system that is battery free and automatically generates electrical stimulations in correlation to stomach movement. A flexible nanogenerator device is developed to be attached to the stomach surface and produce biphasic electrical pulses in response to the peristalsis of stomach. The electric signals can stimulate the vagal afferent fibers to reduce food intake and eventually achieve weight control. We successfully demonstrated this strategy on rats and achieved 38% weight loss in as short as 15 days without further rebound, exceeding all current electrical stimulation approaches. This work provided an effective weight control strategy that is self-responsive, battery free, and directly correlating food intake to stomach movements.

## Results

### Development and working principle of the VNS device

**Fig. 1:**
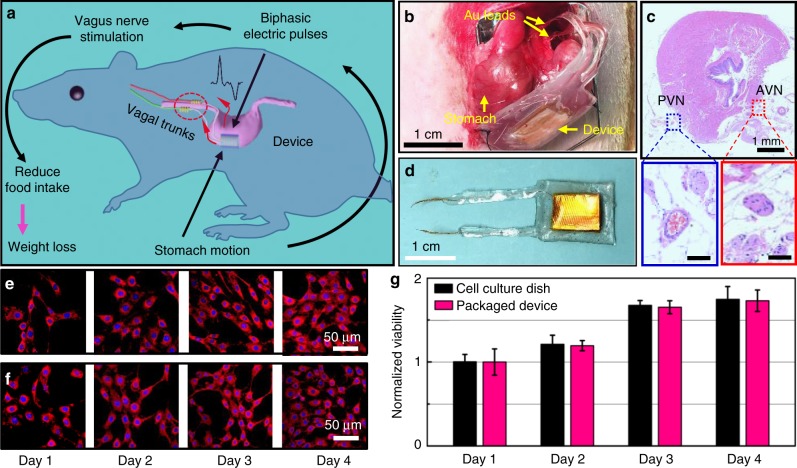
The correlated vagus nerve stimulation (VNS) system and its biocompatibility. **a** Operation principle of the correlated VNS system schematically showing the pathway for biphasic electric signal generation and VNS. **b** An implanted VNS device with Au leads being connected to anterior and posterior vagal trunks. **c** Hematoxylin–eosin (H&E) staining of the implanted tissues (transverse section). Areas within the blue and red boxes are enlarged view of the anterior (red) and posterior (blue) vagus nerves (scale bar = 100 μm). **d** A packaged VNS device. **e**, **f** Fluorescence images of stained 3T3 cells that were cultured on a regular cell culture dish (**e**) and on the surface of a packaged device (**f**). **g** Comparison of normalized cell viability for 4 days showing excellent biocompatibility of the packaged device (*n* = 3 for each group). All data in **g** are presented as mean ± s.d.

The correlated VNS system for weight control is designed following the principle depicted in Fig. 1a. The stomach motion is used as the sole source to generate pulsed voltage signals, which in response will stimulate the vagus nerves to reduce food intake. This self-responsive function is enabled by a triboelectric nanogenerator (TENG)^27–31^ attached on the surface of stomach, which generates biphasic electric pulses when the stomach is in peristalsis. Here, a bilateral VNS is implemented by wrapping the two gold (Au) leads around the anterior vagus nerves (AVNs) and posterior vagus nerves (PVNs) at the proximity of the gastroesophageal junction (Fig. 1b). The AVNs and PVNs were ~6 mm apart and could be clearly observed via multiple staining images (Fig. 1c, Supplementary Figure 1). Connecting at this position could provide a focused stimulation to the small unmyelinated C fibers and avoid stimulating fibers that join the trunk from the heart and lungs^32^.

To ensure the mechanical robustness and flexibility of implanted devices and to avoid potential erosion in the physiological environment, the entire VNS device was packaged by a multilayer film composed of polyimide, polydimethylsiloxane (PDMS), and ecoflex. Au leads were connected to the tips of Cu wire electrodes and partially exposed for electrical signal transmission (Fig. 1d, fabrication details are included in Supplementary Figure 2a and b, which was described in Supplementary Note 1). The TENG was able to generate reasonably high voltage and current output under normal contact-separation motions, with an optimal output power of ~40 µW at an external load of 20MΩ (Supplementary Figure 2c, d and e). Based on the impedance of the vagus nerve, the stimulation voltage was found to be around 200 mV (Supplementary Figure 2d). Similar outputs were obtained from various displacement motions, suggesting that the TENG was able to respond to complex stomach motions (Supplementary Figure 3). To confirm the biocompatibility of the packaged VNS device, mouse fibroblast 3T3 cells were cultured on the encapsulated device surface and in a reference cultural dish for 4 days to examine and compare the cell attachment, proliferation, and morphology. Cells in both media exhibited similar density and equivalent morphology. No dead or distorted cells were observed from the encapsulation material surface (Supplementary Figure 4). The fluorescent staining results showed that the 3T3 cells can spread and form intact cytoarchitecture in both groups (Fig. 1e, f). In addition, 3-{4,5-dimethylthiazol-2-thiazolyl}−2,5-diphenyl-2*H*-tetrazolium bromide (MTT) assay revealed that the relative viability of 3T3 cells on encapsulation material was more than 98% within 4 days, comparable to the cells cultured in the culture dish **(**Fig. 1g). These results confirmed that the encapsulated device is non-cytotoxic and biocompatible.

**Fig. 2:**
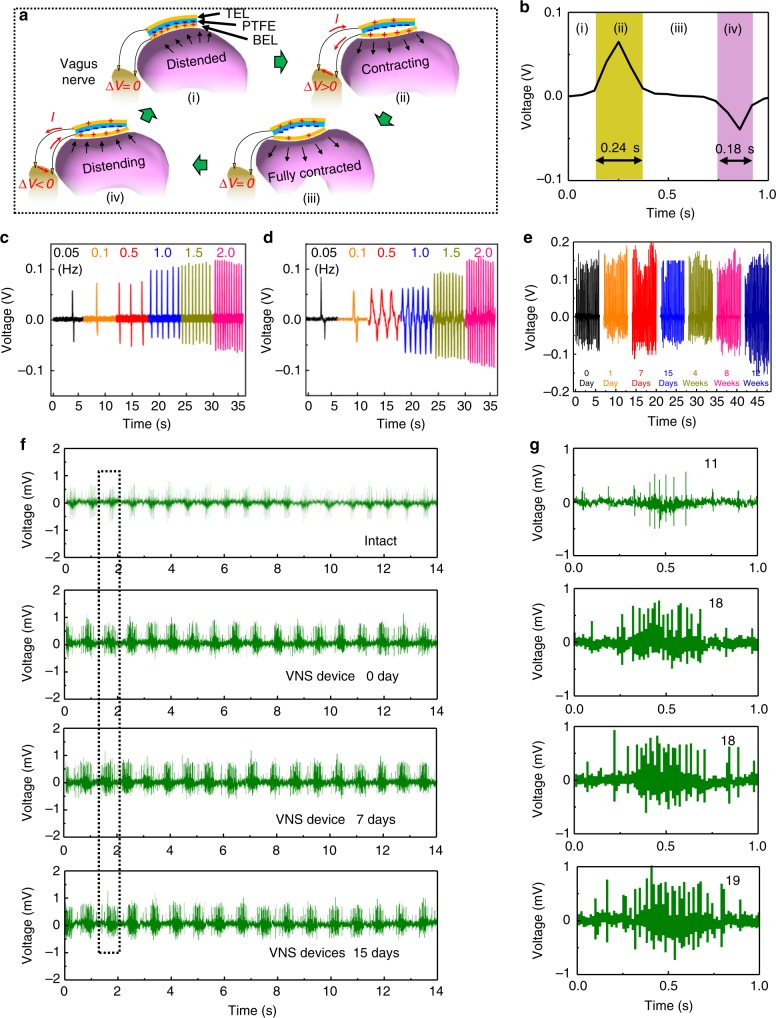
Working principle and voltage signal of the vagus nerve stimulation device. **a** Schematics of the working principle of VNS device under different stomach motion stages. **b** A typical single-cycle voltage biphasic signal corresponding to the four stages of stomach movement at a frequency of 0.05 Hz. **c** Voltage signal measured in PBS solution under different agitation frequency when the VNS device was connected to an external load with the same impedance of the implanted area. **d** Voltage signal measured when the VNS device was implanted and connected to vagus nerves. **e** Long-term stability test of the VNS device, where the device was removed from the rats and the voltage was measured on an external load of 0.3MΩ. **f** Electrophysiological signals recorded from rats without implantation and with an active implanted VNS device on the same day, and 7 and 15 days post implantation. **g** Enlarged view of one group of electrophysiological signal highlighted in the dotted box in **f**

When the stomach is under peristalsis stomach^33,34^, the corresponding motion cycle of the triboelectric layers in the VNS device is depicted in Fig. 2a (i)–(iv). As the stomach is distended, the two triboelectric layers are pushed into contact, where oppositely charged surfaces are created based on their different electron affinity (stage i). The following contraction of the stomach pulls the bottom electrode layer (BEL) layer away from the polymer layer, and thus drives electrons flowing from the top electrode layer (TEL) electrode to the BEL through the two connections with the vagus nerve (stage ii). When the stomach is fully contracted, the triboelectric layers are fully separated, where maximum charge transfer is reached and the net current through the nerve drops back to zero (stage iii). The BEL layer is then brought back toward the polymer layer in the following stomach distention (phase iv), resulting in an opposite current flow through the vagus nerve until the stomach reaches the original distended stage (i) again. The recorded voltage output within one cycle at the frequency of 0.05 Hz is shown in Fig. 2b and corresponding stages are marked along the curve. This contributes to the cyclic alternating electrical signals as the stomach continues peristalsis.

To investigate how the implanted VNS device functioned in response to stomach movements, voltage signals were first measured between the two Au leads. The stomach was arbitrarily deformed by cyclically pressurizing it via a gavage needle at a series of frequencies of 0.05, 0.1, 0.5, 1.0, 1.5, and 2.0 Hz (Fig. 2c). Correspondingly, the voltage signals were also measured when the two Au leads were connected to the vagus nerve in a rat’s body (Fig. 2d and Supplementary Movie 1). Both voltage signals exhibited similar amplitudes ranging from 0.05 to 0.12 V. It should be noted that the recorded voltage was lower than the actual operational voltage due to the finite internal impedance of the measurement system (1MΩ). Higher voltage outputs were recorded from higher frequency. While the stomach deformations (i.e., pressure change) remained constant, higher voltage signal could be attributed to a higher displacement rate, suggesting faster stomach motion is favorable for more intense stimulation. The implanted devices were removed from the rats 1 day, 7 days, 15 days, 4 weeks, 8 weeks, and 12 weeks after implantation, and their voltage output was measured accordingly. All the devices showed a good structural integrity without any observable defects (Supplementary Figure 6). The nearly unchanged voltage amplitude confirmed good stability and durability of the VNS device in the biological environment (Fig. 2e).

Electrophysiological signals were measured from the cervical vagal trunk on the same day, and 7 and 15 days after implantation (Supplementary Figure 7). As shown in Fig. 2f, when there was no external stimulation, a regular electrophysiological signal from the vagus nerve can be detected. The voltage amplitude was ~0.5 mV with 11 electric pulses per signal group (Fig. 2g). When the VNS device was activated, the amplitude increased to ~0.8 mV, and the number of electric pulse per group increased to 18–19. This measurement clearly showed that the VNS device can stimulate vagus nerves effectively. Similar electrophysiological signals could be detected at different time points post implantation, which evidenced the vagus nerves were effectively stimulated by the VNS device during the implantation period. Electrophysiological signals were further measured in response to a range of stimulation voltage from 50 to 740 mV. The stimulated state of the vagus nerve was detected as the voltage from VNS device was above 100 mV. The signal intensity from the vagus nerve increased monotonically following the stimulation voltage (Supplementary Figure 8, Supplementary Note 2).

### Biocompatibility and biosafety of implanted VNS device

**Fig. 3:**
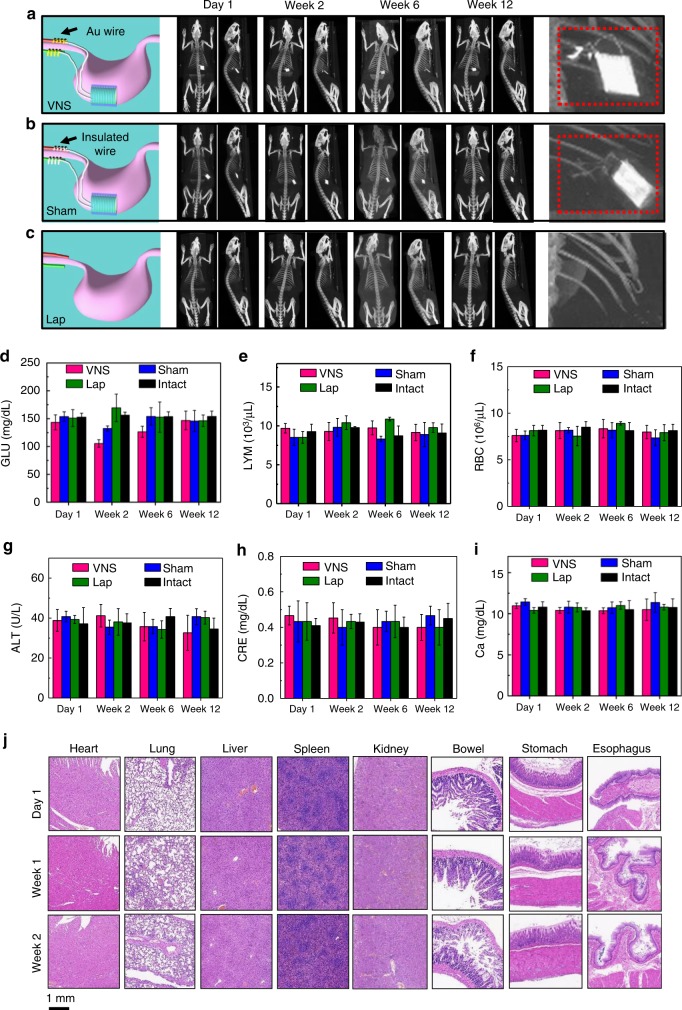
Computed tomography (CT) 3D projection images and hematology data. **a**–**c** Serial CT images over time of the VNS group, Sham group, and Lap group, respectively. Schematics on the left show the setup of each group. A series of CT images (coronal and sagittal) show a representative rat for each group at different time points. The enlarged views of the implantation site are shown at the end. **d**–**i** Hematology results of all four groups over time (*n* = 3 for each group). **d** Blood glucose (GLU) levels. **e** Infection-related lymphocytes (LYM) levels. **f** Hematopoietic function-related red blood cell (RBC) levels. **g** Hepatological function-related alanine aminotransferase (ALT) levels. **h** Renal function-related creatinine (CRE) levels. **i** Electrolyte metabolism-related calcium (Ca) levels. **j** H&E stains of vital organs (heart, lung, liver, spleen, kidney, bowel, stomach, and esophagus) at different time points (1 day, 7 days and 15 days) post implantation. All data in **d**–**i** are presented as mean ± s.d.

Rats with the VNS device implanted on stomach and the Au leads connected to the vagus nerve were defined as the VNS-active group. Small animal computed tomography (CT) was used to produce three-dimensional (3D) x-ray images of representative rat models as a function of implantation time to investigate the implantation stability when the rat was under normal daily activity (Fig. 3a, Supplementary Figure 9, and Movie 2). The high contrast spot (Au is a good contrast agent for CT) inside the rat was the implanted VNS device. No position shifting was observed during the entire 12 weeks of implantation period. This high stability could be attributed to the good biocompatibility of the packaged VNS device, which was observed being completely imbedded possibly by omentum and tightly fixed to the stomach surface post study (Supplementary Figure 10). The right panel of Fig. 3a shows an enlarged CT image of the implantation area, where the Au leads and exposed tip (with much brighter contrast) can be clearly identified, wrapping around at the vagus nerve region. In contrast to the VNS group, the sham group had the same VNS device implanted on stomach but without Au leads connecting the Cu wire electrodes to the vagus nerve (the completely packaged Cu wires were still placed at the same vicinity of the vagus nerve, Fig. 3b). 3D CT images showed the same stable VNS implantation in the sham group. The enlarged image revealed the insulated leads exhibiting a uniformly low contrast. As a comparison, rats in the laparotomy (Lap) group, which rats were subjected to surgery but without the VNS device implantation, were also imaged and showed only the skeleton of the rats (Fig. 3c). The Sham, Lap, and Intact (rats without any operation) groups are defined as the control groups.

Whole blood and chemical analysis were performed on the four groups of rats for biosafety assessment during the implantation period. Compared to the hematology data in intact group, the blood glucose (GLU) concentration (Fig. 3d) in VNS and Sham group decreased at week 2 due to reduced food intake after surgery, and eventually recovered to the normal levels. The indicators of infection such as lymphocytes (Fig. 3e), hematopoietic function such as red blood cell (RBC) (Fig. 3f) and hemoglobin (Supplementary Figure 11a), hepatological function such as alanine aminotransferase (ALT) (Fig. 3g) and albumin (Supplementary Figure 11b), renal function such as creatinine (Fig. 3h) and blood urea nitrogen (Supplementary Figure 11c), and electrolyte metabolism such as calcium (Ca) (Fig. 3i) and phosphorus (Supplementary Figure 11d) all remained steady during the entire implantation period. In general, all the blood testing results were within the normal range shortly after the device implantation and did not show any abnormality^35,36^, suggesting that the VNS device is highly hemocompatible. The comprehensive blood analyses, together with the imaging results, confirmed that implanting the VNS device on stomach surface did not cause any measurable adverse effect to the rats. All rats with the VNS implantation exhibited normal daily behaviors, which were not different from the intact groups (Supplementary Movie 3).

Pathological tests were conducted on most vital organs, including heart, lung, liver, spleen, kidney, bowel, stomach, and esophagus. Hematoxylin and eosin (H&E) staining were collected from these organs at different time points (1 day, 7 days, and 15 days) post implantation. All the organs showed no deformation and abnormal lymphatic cell invasion (Fig. 3j), which further confirmed that all the rats were in a good health condition, and the VNS device had no systemic side effects. Histological analysis of the vagus nerves 15 days after implantation showed no signs of nerve cell shape change or invasion of inflammation cells (Supplementary Figure 12). This revealed the vagus nerves were not damaged by connecting the VNS device.

### Weight control by implanted VNS device

**Fig. 4:**
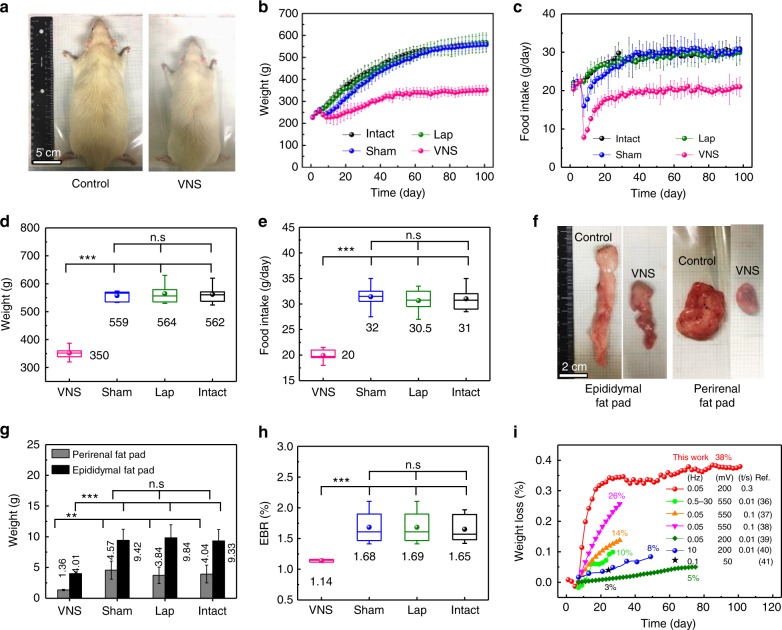
Weight control during the weight-gaining growth stage of rats. **a** Representative images of body size of the VNS group (*n* = 6) and the control groups (Sham, Lap, and Intact group, *n* = 6 for each group). **b** Average body weight of rats in different groups over time (implantation was performed after a week of observation period under normal conditions). **c** Rat’s food intake over time in different groups. **d** Final body weight of rats in different groups. **e** Final daily food intake in steady state of rats in different groups. **f** Representative images of white adipose tissue (epididymal fat pad and perirenal fat pad) of the VNS group and the control group. **g** Adipose tissue weight of rats in different groups. **h** Epididymal fat pad/body weight ratio (EBR) of rats in different groups. **i** Percentage of weight loss over time (black dots) in comparison to the reported results by chronic electric stimulation with a rectangular waveform (voltage, frequency, and pulse duration are also shown). All data in **b**, **c**, and **g** are presented as mean ± s.d. In **d**, **e**, and **h** (box plots), dots are the mean, center lines are the median, box limits are the lower quartile (Q1) and upper quartile (Q3), and whiskers are the most extreme data points that are no more than 1.5× (Q3–Q1) from the box limits. Statistical analysis was performed by two-tailed unpaired Student’s *t* tests. n.s., non-significant (*P* > 0.05); *P < 0.05, **P < 0.01, and ***P < 0.001

The weight control performance was first examined in the four groups of rats (VNS, Sham, Lap, and Intact) that were fed and grown under the same conditions. The average initial weight of rats was 250 g, and their body weight and food intake were monitored on a daily basis. After 100 days, the body size of the VNS group was significantly smaller than all three control groups (Fig. 4a). The recorded body weight and corresponding daily food intake over time are shown in Fig.  4b, c, respectively. Since the implantation surgery was conducted on the seventh day of this study, all four groups exhibited the same body growth trend and the same amount of food consumption over the first week, indicating that all rats were under the identical growth conditions and their results were comparable. Immediately after the surgery, the VNS and Sham groups (both had VNS implanted) exhibited an obvious weight loss. Accordingly, their food intake was also largely reduced likely due to the VNS device attachment. The Lap group, which had the same surgical procedure/opening, did not show any abnormity in weight change or food intake when compared to the intact group, suggesting that surgery itself had minimal impact on weight control. As the rat’s body adapted to the VNS device implantation, the amount of food intake of the Sham group quickly recovered to the same level as the other two control groups after ~15 days of implantation. As a result, the average body weight of the Sham group bounced back after the initial 2–3 days, and increased following the same trend of the other two control groups (Lap and Intact). After 60 days, all three control groups exhibited a very close body weight of 535 ± 18 g (Sham), 538 ± 32 g (Lap), and 538 ± 32 g (Intact) (*n* = 6 for each control group), confirming that neither surgery nor simple stomach attachment had any effect on weight control. On the contrary, although the food intake of the VNS group recovered as well after the initial reduction and reached a steady level after ~15 days, the daily consumption of food was only ~2/3 of those consumed by the other control groups. Therefore, the average body weight of the VNS group exhibited a much slower growth rate. It reached a steady value of 350 ± 23 g (*n* = 6), about 63% of the other three control groups. Box plots were implemented to provide a statistic analysis of the final body weight (on the day of sacrifice, Fig. 4d) and food intake (on the last day before euthanasia, Fig. 4e). The differences between the VNS group and three control groups were statistically significant (*P* < 0.001) for both body weight and food intake, while the differences between all three control groups were not (*P* > 0.2). Such comparison clearly revealed that spontaneous nerve stimulation by the implanted VNS device had obvious impact on weight control.

All the rats were sacrificed after the 100-day weight control study for anatomical examinations. Similarly, the anatomical adipose tissues (epididymal fat pad and perirenal fat pad, representative of the body fat level)^37^ in the VNS group were significantly smaller than the control groups (Fig. 4f). The average weight of the epididymal fat pad and perirenal fat pad were only 4.01 and 1.36 g, 58 and 67% smaller than the control groups, respectively (Fig. 4g). The epididymal fat pad/body weight ratio (EBR) was calculated by dividing the fat pad weight by the total body weight. Average EBR was maintained at 1.14% in the VNS group, significantly lower than the control groups which all exhibited EBR of ~1.7% (Fig. 4h). By comparing the weight difference between the VNS and control groups, our correlated VNS system rapidly achieved 35% weight loss within 18 days and maintained a weight-loss ratio as high as 38% for remaining study period (75 days), which largely exceeded other reported electrical stimulation approaches based on similar rat models^38–43^ (Fig. 4i).

**Fig. 5:**
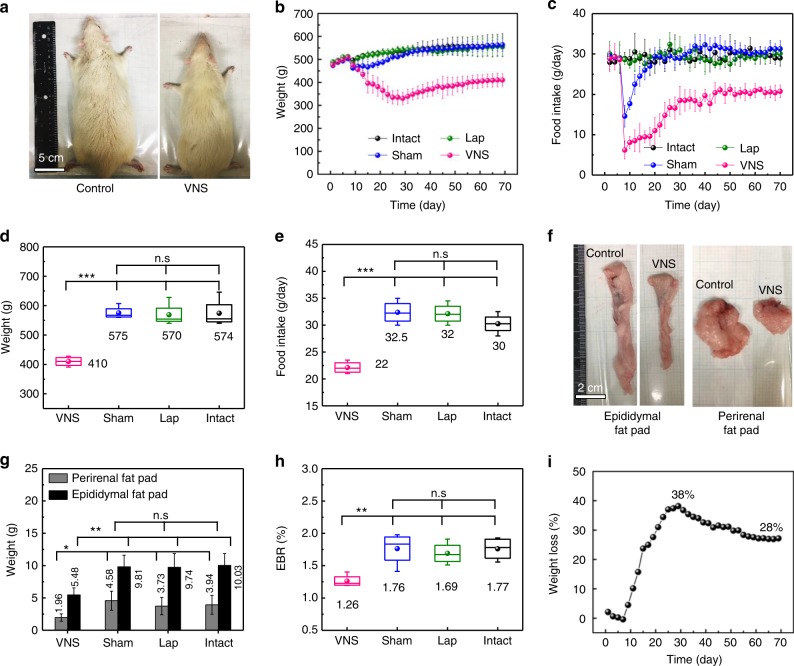
Weight loss of fully grown adult rats after implantation of the VNS device. **a** Representative images of rats in the VNS group (*n* = 4) and the control groups (*n* = 4 for each control group). **b** Average body weight of rats in different groups over time (implantation was performed after 7 days of observation under normal conditions). **c** Rat’s food intake in different groups over time. **d** Final body weight of rats in different groups. **e** Final daily food intake in steady state of rats in different groups. **f** Representative images of white adipose tissue (epididymal fat pad and perirenal fat pad) of the VNS group and the control group (scale bar = 2 cm). **g** Adipose tissue weight of rats in different groups. **h** Epididymal fat pad/body weight ratio (EBR) of rats in different groups. **i** Percentage of weight loss over time after implantation of the VNS device. All data in **b**, **c**, and **g** are presented as mean ± s.d. In **d**, **e**, and **h** (box plots), dots are the mean, center lines are the median, box limits are the lower quartile (Q1) and upper quartile (Q3), and whiskers are the most extreme data points that are no more than 1.5× (Q3–Q1) from the box limits. Statistical analysis was performed by two-tailed unpaired Student’s *t* tests. n.s., non-significant (*P* > 0.05); *P < 0.05, **P < 0.01, ***P < 0.001

To further exploit the capability of the VNS system for weight loss, the same implantation surgery and analyses were conducted on grown adult rats that have been fed for 7 weeks and reached a steady average body weight of ~500 g. Little body weight gain was observed for the intact group during the 70-day study period, while the VNS group exhibited an obvious body size reduction (Fig. 5a). Similar as the previous study, the Lap group exhibited no difference when compared to the intact group, while the Sham group quickly recovered to the same levels after the initial drop in both body weight and food intake (Fig. 5b, c, respectively). Food intake of the VNS group exhibited a much slower recovery rate and eventually remained at the steady value that was ~2/3 of the control groups. Accordingly, the average body weight of the VNS group exhibited a steep drop over the first 25 days after implantation, followed by a small recovery and stabilized at ~400 g. The final body weight (Fig. 5d) and food intake (Fig. 5e) showed significant differences between the VNS group and control groups, and no significant difference were found among the control groups. The final average body weight was controlled at 410 ± 17 g (*n* = 4), significantly smaller than the control groups (Sham: 575 ± 22 g; Lap: 569 ± 39 g; and Intact: 574 ± 48 g, *n* = 4 for each control group). Significant differences were also found in the adipose tissue sizes (Fig. 5f) and weight (Fig. 5g). The final EBR was controlled at 1.26% in the VNS group, while the three control groups maintained a much high value from 1.69% to 1.77% (Fig. 5h). The calculated weight-loss percentage peaked at 38% at day 29 and gradually reached a stable 28% (Fig. 5i).

## Discussion

In this work, we present a correlated VNS system as an effective therapeutic strategy for obesity, which provided correlated nerve stimulation signal in response to stomach peristalsis. The VNS device was built based on a flexible TENG that was attached to the stomach wall of rats and could generate biphasic electric pulses when the stomach wall moved. The TENG electrodes were directly connected to the vagus nerve, where the stomach motion-generated voltage signals stimulated the vagus nerve to reduce food intake. We envision that this correlated stimulation could provide less amount but more targeted stimulation so that the nerves might be more responsive to the stimulation, and thus more effective to control food intake. The VNS device exhibited excellent biocompatibility without any signs of side effects from the whole blood and chemical analysis. CT and hematology indicators revealed the implantation was very stable and remained at the same position during the entire testing period. The weight control performance was examined and compared among the VNS, Sham, Lap, and Intact groups of rats that were fed and grown under the same conditions. From the weight-gain test, the average body weight and EBR can be controlled at 350 g and 1.14% in the VNS group, compared to 559–564 g and 1.65–1.69% in the other three control groups with insignificant differences. The VNS system rapidly achieved 35% weight loss within 18 days, which was maintained 38% during the remaining 75-day testing period. From the weight-loss test, the average body weight and EBR was controlled at 410 g and 1.26% in the VNS group, compared to 570–575 g and 1.69–1.77% in the other three control groups. Rats in the VNS groups were also able to recover their normal weight pattern immediately after the implanted VNS devices were removed (Supplementary Figure 13, Supplementary Note 3). Our correlated VNS system demonstrates outstanding weight control results, which largely outperformed other reported chronic microchip VNS systems based on similar rat models. In addition, this correlated VNS is battery free and less invasive compared to the bariatric surgical strategies (e.g., gastric bypass, biliopancreatic diversion or sleeve gastrectomy) for weight control. For future clinical trials, a switch may be needed by the VNS device to control the treatment. This could be achieved by integrating a shutter switch to the electrical wires from the VNS device, as it has been proved that a disconnected device has no impact to food intake and weight change. More broadly, this development demonstrated a successful example of a self-responsive and real-time peripheral neuromodulation mechanism that may be more effective for achieving therapeutic purpose.

## Methods

### Device fabrication and encapsulation

Polyimide film (50 μm) was used as the core package layer that was proven to be biocompatible and corrosion resistant for bio-implanted devices^44,45^. Casting and curing a pre-polymer to PDMS (15:1 PDMS; Dow Corning, USA) covers the entire device as the shell package (1 mm) to enhance the leakproof and ensure good structural flexibility and stability. To further increase the flexibility to closely fit the non-planar surfaces of stomach and maintain sensitivity in response to stomach motions, a layer of ecoflex (200 μm) was coated onto the surface as another shell structure of the device^46–48^. The PTFE surface was treated by reactive ionic etching to introduce nanostructured features to enhance the electrical output^49^ (Supplementary Figure 2a). The overall VNS device dimensions are approximately 16 × 12 × 2.5 (*L* × *W* × *T*) mm^3^ and the weight was measured to be only ~0.8 g.

### Electrical characterization of VNS devices

The electrical performance of all implanted VNS devices were measure by a portable oscilloscope (Agilent, DSO1012A, internal impedance is 1MΩ). The voltage signals shown in Fig. 2b, d were measured directly from an implanted VNS device where the two Au leads were connected to the vagus nerve. The stomach motions were induced by injecting water into the stomach using a gavage needle through the mouth and the injection volume difference was 2 mL. The voltage signal shown in Fig. 2c was measured by pressing the VNS devices in phosphate-buffered saline (PBS) solution under different frequency when the VNS device was connected to an external load with the same impedance of the implanted area. Voltage signals in Fig. 2e and Supplementary Figure 3 were measured by the pressing the VNS devices at frequency of 4 Hz when connected to an external load of 0.3MΩ, the same as the impedance of the vagus nerve. The voltage and current output of TENGs as a function of load (10Ω to 200MΩ) was measured by a Stanford Research Systems model SR 560 low-noise preamplifier (internal impedance is 100MΩ). The impedance of implanted VNS device was characterized from 0.01 to 10,000 Hz using an Autolab PGSTAT302N station (Supplementary Figure 5).

### Electrophysiological properties of vagus nerve

A Sprague–Dawley rat was anesthetized and its right cervical vagal trunk was carefully exposed (Supplementary Figure 7a). A pair of bipolar platinum hook electrodes was then placed under the right vagal nerve immediately. The exposed nerve tissue was covered with warm (37 °C) paraffin oil. The electrical signals were recorded and analyzed by a Cambridge Electronic Design (CED) 1401 interface (Cambridge, UK) with Spike 2 software to monitor the change the electrical signal of the VNS device (Supplementary Figure 7b and c). The VNS device was implanted in rat’s body with Au leads being connected to anterior and PVNs. The implanted VNS devices were activated by gently pressing the abdomen of the rats at a frequency of 1 Hz. In addition, VNS devices with different sizes were fabricated to study the response of vagus nerve in corresponding to the amplitude of stimulation voltage (Supplementary Figure 8).

### Animals and diets

All animal experiments were conducted under a protocol approved by the University of Wisconsin Institutional Animal Care and Use Committee. Seven- and eight-week-old male Sprague–Dawley rats were acquired from Envigo (New Jersey, USA). All rats were housed in separated cages at a temperature-controlled room (22 °C) with a 12-h light/dark cycle with free access to water and Purina PMI-certified rodent chow 5002 (LabDiet, MO, USA).

### Food intake and body weight

Body weight and food intake was recorded at 8:00 p.m. every other day. The daily food intake was determined from the difference in food quality between each measurement and divided by two. All rats were deprived of food for 12 h before surgical implantation and blood test. Percentage of weight loss (*P*_weight loss_) over time in Figs. 4i and 5i was calculated according to the formula: *P*_weight loss_ = (*W*_Intact_–*W*_VNS_)/*W*_Intact_ × 100%, where *W*_Intact_ and *W*_VNS_ represent the average weight of the Intact group and the VNS group, respectively.

### Histological staining of vagus nerve and vital organs

Tissue slices of the bottom of esophagus and its surrounding tissue were prepared. H&E staining, immunohistochemical (Supplementary Figure 1a), and immunofluorescent (Supplementary Figure 1b) staining using anti-S-100 rabbit anti-rat poly-antibody^50^ showed clearly the anterior vagal trunk and posterior vagal trunk distributed on both sides of the esophagus. Vital organs including heart, lung, liver, spleen, kidney, bowel, stomach, and esophagus were retrieved from rats for H&E staining after euthanasia at different time points (1 day, 7 days, and 15 days) post implantation. In addition, vagus nerves were re-evaluated after the device being implanted for 15 days, and the additional H&E staining results were shown in Supplementary Figure 12.

### Cell morphology and immunofluorescence staining

After 3T3 cells were cultured on encapsulation or cell plates in 24-well plates, cell morphology was observed directly using an inverted optical microscope (Nikon Eclipse Ti-U, Japan). The cytoskeleton and nucleus were stained with Texas Red-X phalloidin (591/608 nm) and blue fluorescent Hoechst (352/461 nm) (Thermo Fisher Scientific), respectively. The samples were fixed with 2–4% formaldehyde for 15 min and then rinsed three times with pre-warmed PBS. The samples were incubated with Texas Red-X phalloidin (100 nM) and Hoechst (50 nM) for 30 min at 37 °C. After staining, cells were rinsed with pre-warmed buffer for three times and imaged using a Nikon A1RS confocal microscope.

### MTT assay

After 3T3 cells were cultured on the packaging film on 24-well plates, MTT assay (Thermo Fisher scientific) was performed to examine cell proliferation. After incubation at 37 °C in a humidified atmosphere with 5% CO_2_ for up to 4 days, 100 μL of MTT solution was added to each well. After 4-h incubation, the medium was removed and dimethyl sulfoxide (500 μL/well) was added to dissolve the precipitated fomazan. The optical density (*n* = 3) of the solution was evaluated using a microplate spectrophotometer at a wavelength of 490 nm.

### Device implantation

In brief, anesthesia was induced by inhalation of 2–5% isoflurane and maintained with 2% isoflurane. Following anesthesia, rats were fixed in supine position. The abdomen of rats was scrubbed with iodine scrub, and then alcohol prior to surgery. An incision of 2–5 cm was made on the left upper abdomen of rats. The device was placed beside the stomach. The anterior and posterior vagus nerves were identified and separated from the gastroesophageal junction. For the VNS group, Au wires were placed in contact with the nerves and secured with sterile surgical tape. For the sham group, the device was implanted with insulated electrodes connecting to the vagus nerves. Afterwards, the muscle and skin were sutured layer by layer and the stitches were removed 2 weeks later. The implantation procedure is shown step by step in Supplementary Figure 14. The entire surgery lasted approximately 15 min.

### CT scan

CT whole-body scan was performed to evaluate the position and integrity of the VNS device post implantation, which can generate 3D images and reconstruct a high-definition 2D projection image of rats with a resolution of up to 100 µm. In brief, rats were placed in the prone position after anesthesia and scanned by an Inveon micro positron emission tomography/CT scanner (Siemens Medical Solutions, USA) at 1 day, 2 weeks, 6 weeks, and 12 weeks post implantation. CT images were reconstructed and presented as 3D projection or slices.

### Hematology data

Whole-blood and chemical analysis were performed for safety assessment pre-implantation and 2, 6, and 12 weeks post implantation. Blood was drawn from the tail vein of the rats and various tests were performed using an Abaxis VetScan HM5 Hematology Analyzer (Abaxis, USA) and VS2 Blood Chemistry Analyzer (Abaxis, USA). No centrifugation or other treatment were needed.

### Anatomic examination and adipose tissue collection

After rats were euthanized at the end of the study, an incision was made on the abdomen. The stomach along with the VNS device were taken out for further analysis. In addition, epididymal fat pad and perirenal fat pad were removed and weighed for further analysis (Supplementary Figure 15).

### Statistical analysis

For the final body weight, food intake, and adipose tissue weight, statistical analysis was performed by two-tailed unpaired Student’s *t* tests. n.s., non-significant (*P* > 0.05); *P < 0.05, **P < 0.01, ***P < 0.001. In box plots, dot is the mean, center line is the median, box limits are the lower quartile (Q1) and upper quartile (Q3), and whiskers are the most extreme data points that are no more than 1.5× (Q3–Q1) from the box limits.

## Supplementary Information

Supplementary Information

Description of Additional Supplementary Files

Supplementary Movie 1

Supplementary Movie 2

Supplementary Movie 3

Reporting Summary

## Data Availability

The authors declare that all data supporting the findings of this study are available within the Article and its Supplementary Information. The raw data generated in this study are available from the corresponding author upon reasonable request.
