# Supplementary material for: Effective weight control via an implanted self-powered vagus nerve stimulation device
Source: Nat Commun. 2018 Dec 17;9:5349. doi: 10.1038/s41467-018-07764-z (PMC6297229; doi:10.1038/s41467-018-07764-z)
Supplement: Supplementary file 1 — Supplementary Information [file 41467_2018_7764_MOESM1_ESM.pdf]

Supplementary information for

Effective Weight Control via an Implanted Self-Powered Vagus Nerve Stimulation Device

Guang Yao[1,3†], Lei Kang[2,4†], Jun Li[1], Yin Long[1,3], Hao Wei[2], Carolina A. Ferreira[2], Justin J. Jeffery[5], Yuan Lin[3], Weibo Cai[2]*, Xudong Wang[1]*

[1]Department of Materials Science and Engineering, University of Wisconsin-Madison, Madison, Wisconsin 53706, USA.

[2]Department of Radiology, University of Wisconsin-Madison, WI 53705, USA.

[3]State Key Laboratory of Electronic Thin films and Integrated Devices, University of Electronic Science and Technology of China, Chengdu, Sichuan 610054, P. R. China.

[4]Department of Nuclear Medicine, Peking University First Hospital, Beijing 100034, P. R. China.

[5]University of Wisconsin Carbone Cancer Center, Madison, WI53705, USA

[†] These authors contributed equally to this work.

* Correspondence should be addressed to X.W. (xudong.wang@wisc.edu) or W.C. (wcai@uwhealth.org)

Supplementary Figure 1

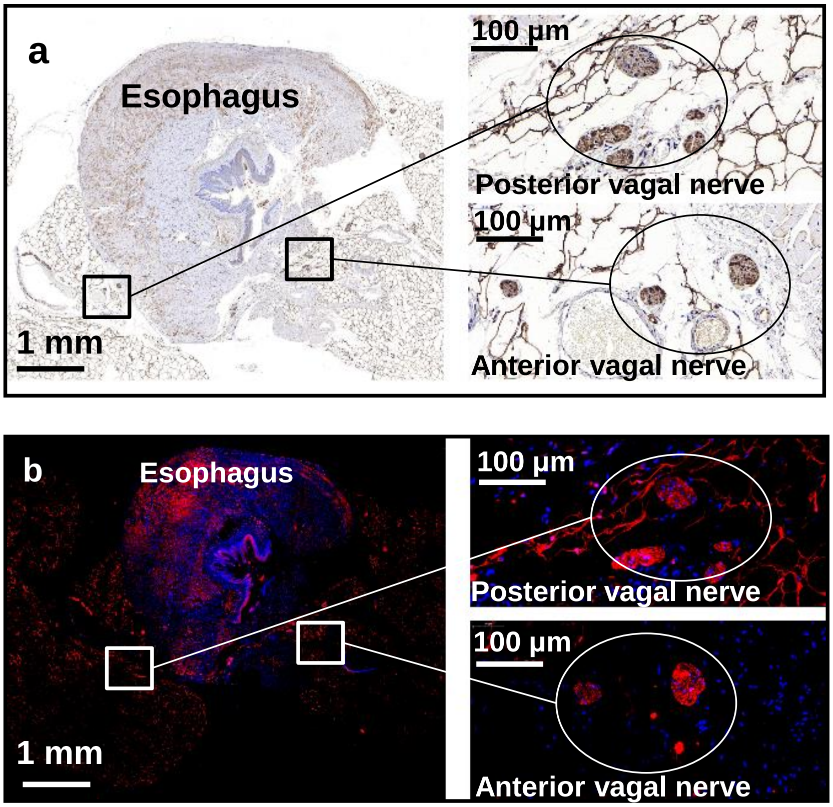

Supplementary Figure 1. Immunohistochemical and immunofluorescent staining. (a) Immunohistochemical and **(b)** immunofluorescent staining images using anti-S-100 rabbit anti-rat poly-antibody showing anterior and posterior vagus nerves.

Supplementary Figure 2

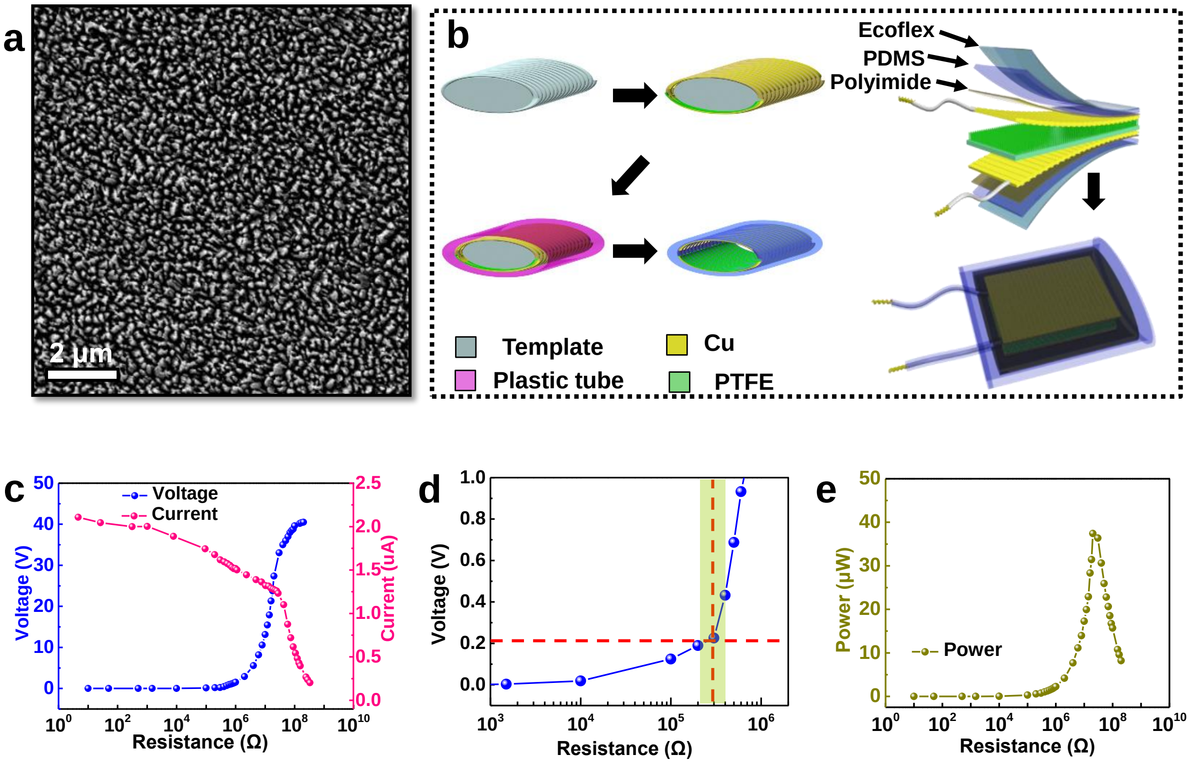

Supplementary Figure 2. Fabrication details and output performance of the device. (a) Scanning electron microscopy image of the nanostructured PTFE surface. **(b)** Schematic fabrication and encapsulation procedures of the VNS device. **(c)** Voltage and current output measured from the VNS device as a function of load resistance. **(d)** Enlarged views of (c). Considering the impedance of the vagus nerve is ~0.3 MΩ, the operational voltage should be in the range of 180-250 mV. **(e)** Output power density as a function of load resistance calculated form the voltage and current data.

Supplementary Figure 3

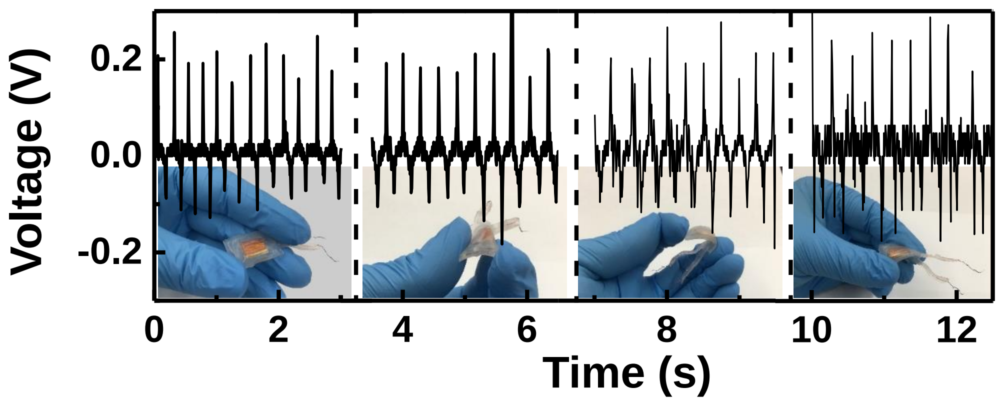

Supplementary Figure 3. **Voltage outputs of various displacement motions.** Electric outputs measured from the VNS device under different movements, including vertical pressuring, length-axis bending, width-axis bending, and diagonal axis bending.

Supplementary Figure 4

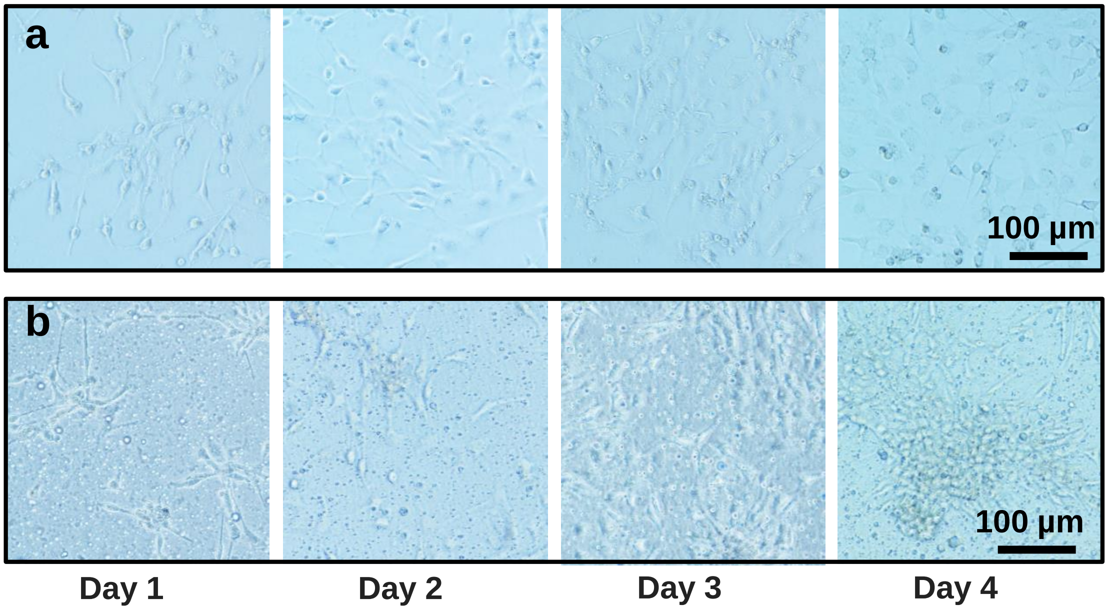

Supplementary Figure 4. Cell morphology evaluation on packed material. Cell morphology evaluation of mouse fibroblast 3T3 cells on a regular cell culture dish **(a)** and on the surface of the packaged device **(b)**.

Supplementary Figure 5

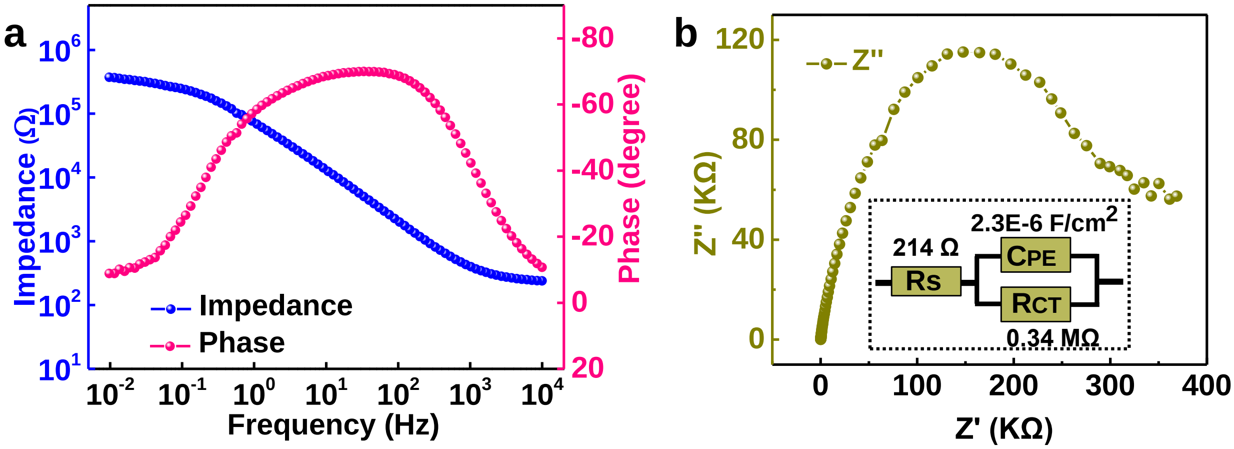

Supplementary Figure 5. Impedance of implanted VNS device. (a) Electrochemical impedance spectroscopy measurements of the vagal tissue were conducted from 0.01 Hz to 10,000 Hz with the standard three-electrode setup using an Autolab PGSTAT302N station. The contact geometric surface area was 0.02 mm^2. The resistance was about 0.3 MΩ (0.05Hz – 2 Hz). **(b)** Nyquist plot of the vagus nerve tissue from 0.01 Hz to 10,000 Hz. Inset represents the equivalent circuit model used to fit measurement results.[1] The equivalent circuit includes a capacitor ($C_{PE} = 2.3\times10^{-6}$), a charge transfer resistance (R_{CT} = 0.34 MΩ), and a serial resistance (R_S = 214 Ω) that corresponds to the surrounding electrolyte solution (PBS solution). The spectrum showed that the resistance of vagal tissue is ~0.3 MΩ (0.05Hz – 2 Hz).

Supplementary Figure 6

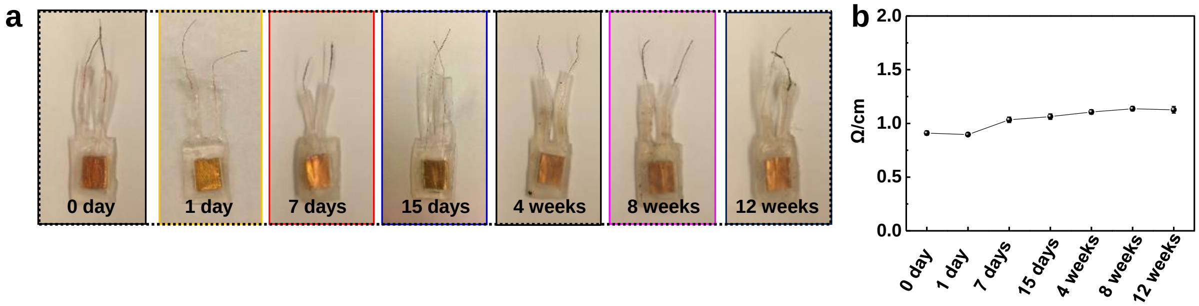

Supplementary Figure 6. Evaluation of device structural integrity. (a) Representative photos of the implanted devices, when removed from the implantation site at different time points. **(b)** Impedances measured from the exposed Au leads from the corresponding VNS devices at different time points. The impedance remained at a stable value of ~1.0 Ω/cm.

Supplementary Figure 7

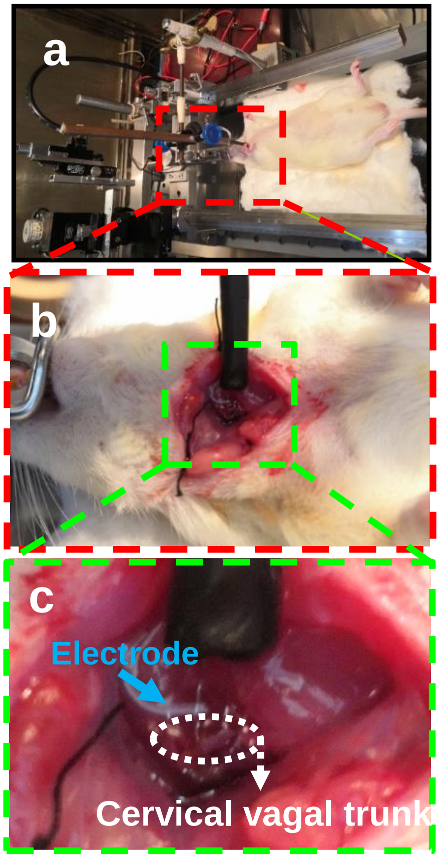

Supplementary Figure 7. Experimental procedure of electrophysiological measurement. (a) A SD rat was anesthetized and its right cervical vagal trunk was carefully exposed. **(b)** A pair of bipolar plantinum hook electrodes was placed under the cervical vagal trunk. **(c)** Enlarged view to show the relative positions of the electrode and vagal trunk.

Supplementary Figure 8

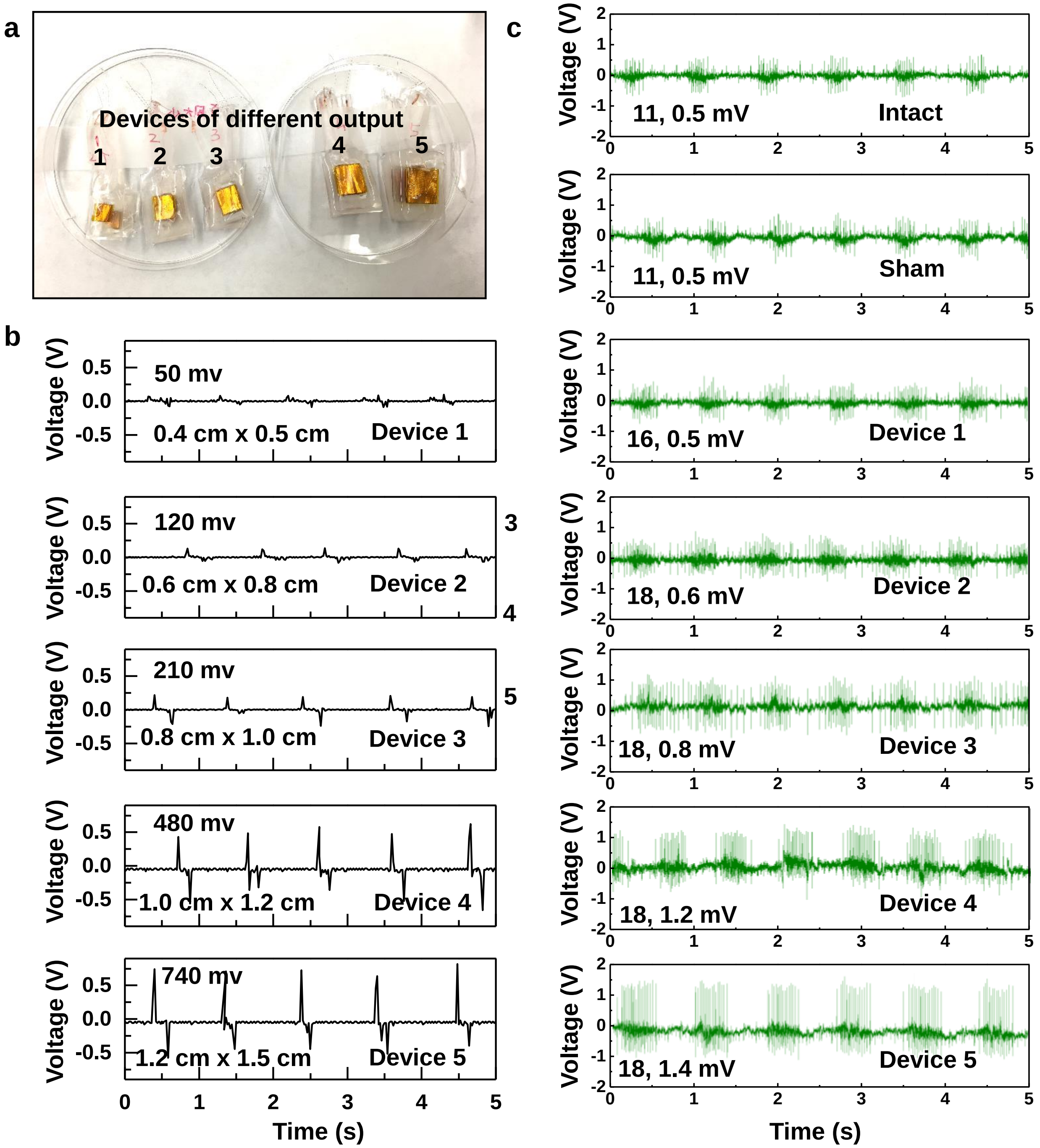

Supplementary Figure 8. Electrophysiological signals from different devices. (a) Photo of VNS devices made with different sizes. **(b)** Voltage output measured from the devices with different sizes. **(c)** Electrophysiological signals under stimulation of different VNS voltage amplitudes.

Supplementary Figure 9

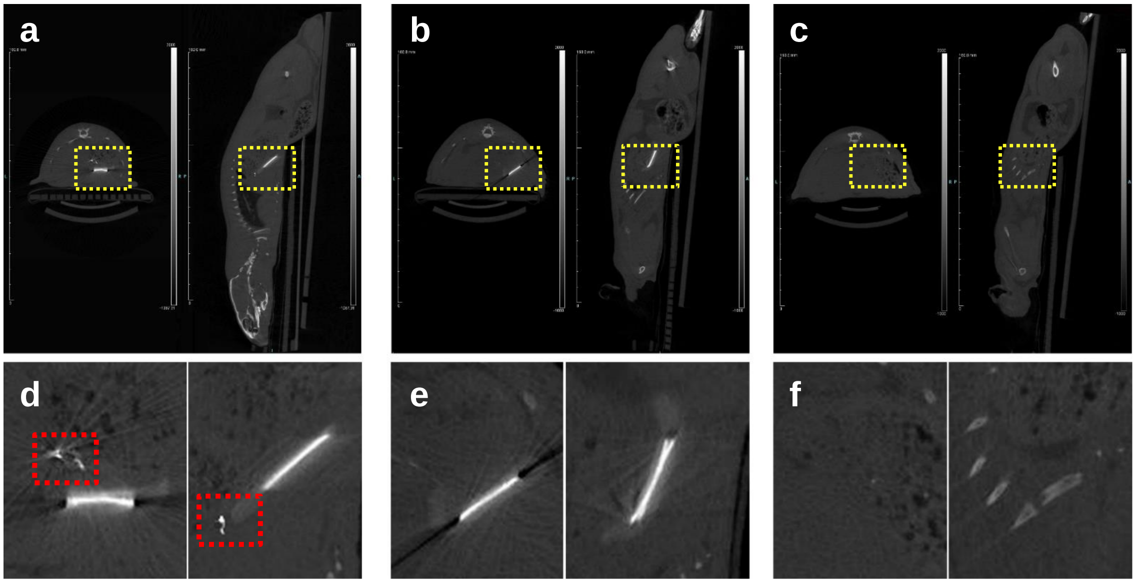

Supplementary Figure 9. Observing implanted devices by CT. (a-c) Transaxial and coronal CT images of rats in the VNS group (a), Sham group (b) and Lap group (c). **(d-f)** Enlarged views of the VNS implantation sites in (a-c), respectively. The high-contrast wire in (**Supplementary Figures 9a and d**) is the exposed Au leads; while the completely packed Cu wires exhibited a lower contrast (**Supplementary Figures 9b and e**); there is no wire in (**Supplementary Figures 9c and f**).

Supplementary Figure 10

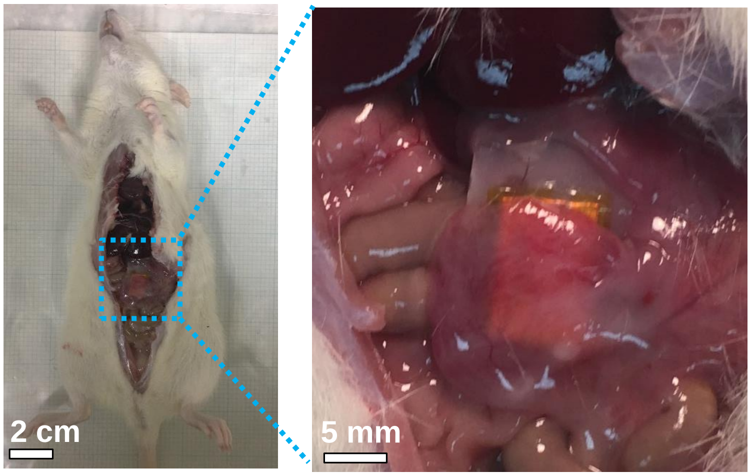

Supplementary Figure 10. Device was fixed to the stomach surface post-study. The implanted

VNS device that was completely imbedded by omentum.

Supplementary Figure 11

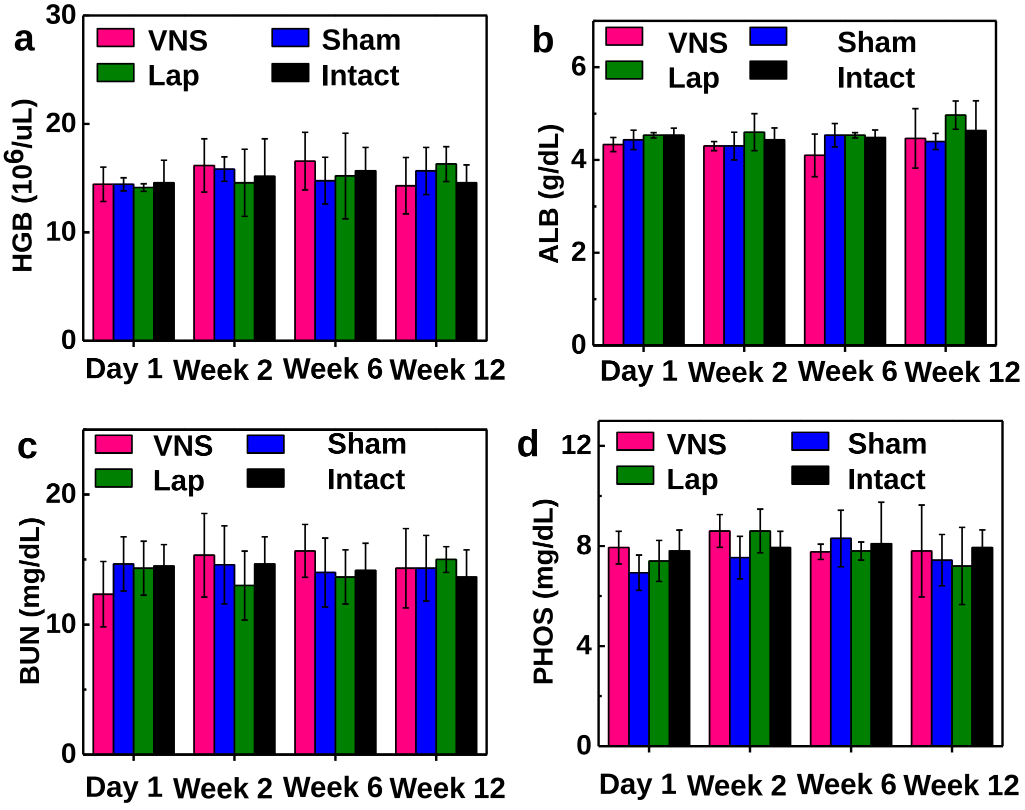

Supplementary Figure 11. Hematology data of different groups over time. (a) Hematopoietic function-related hemoglobin (HGB) levels. **(b)** Hepatological function-related albumin (ALB) levels. **(c)** Renal function-related urea nitrogen (BUN) levels. **(d)** Electrolyte metabolism-related phosphorus (PHOS) levels. All the hematology data were within normal limits, which confirmed that the implanted devices were safe without any toxicity in living rats. All data are presented as mean ± s.d. (n=3 for each group)

Supplementary Figure 12

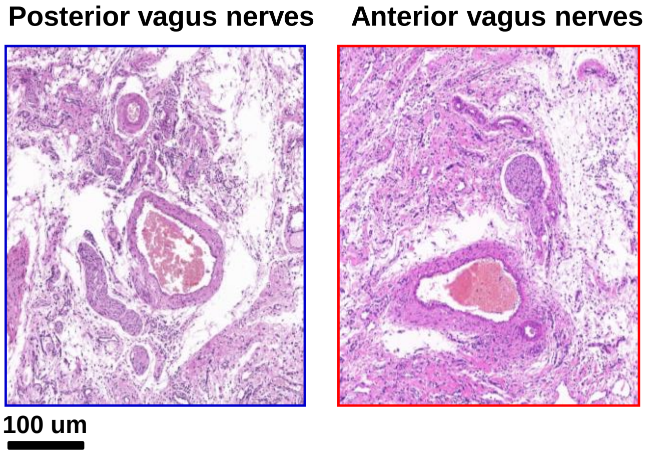

Supplementary Figure 12. Histological analysis of the vagus nerves. Hematoxylin-Eosin (H&E) staining of anterior and posterior vagus nerves after 15-days of VNS device implantation.

Supplementary Figure 13

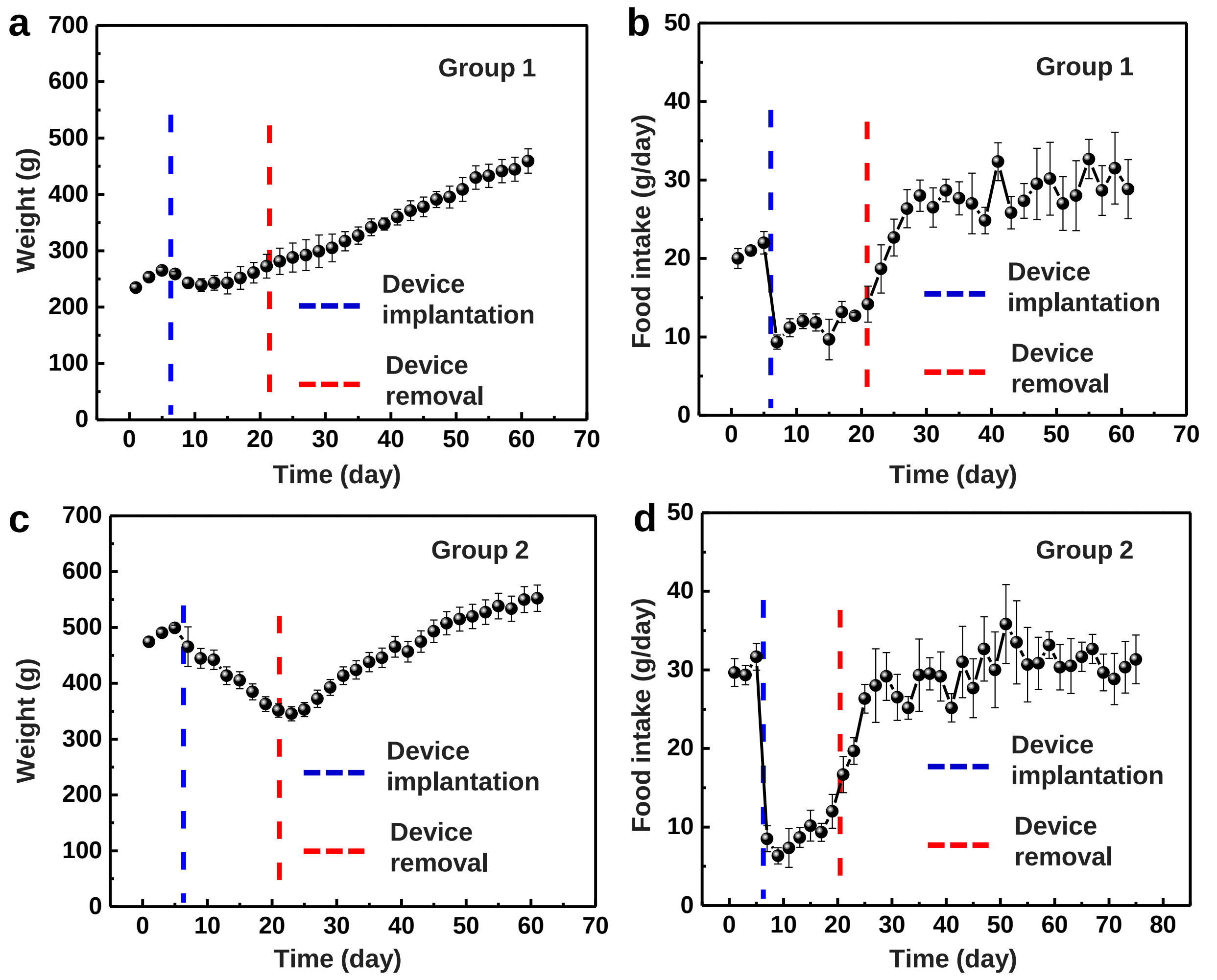

Supplementary Figure 13. Evaluation of body weight before and after the device removal. (a) Average body weight of rats in the weight-gaining group (group 1). **(b)** Average amount of food intake in group 1. **(c)** Average body weight of rats in the fully-grown adult group (group 2). **(d)** Average amount of food intake in group 2. All data are presented as mean ± s.d. (n=3 for each group)

Supplementary Figure 14

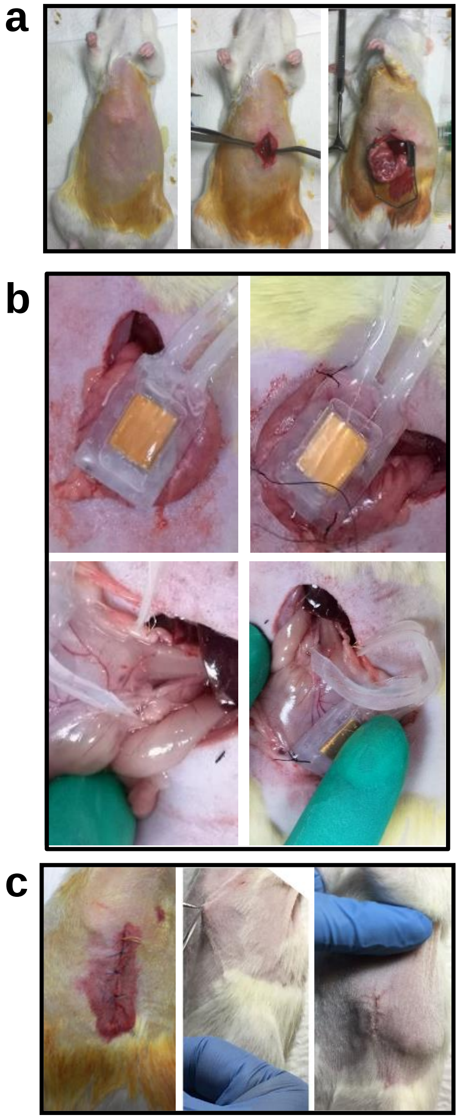

Supplementary Figure 14. Implantation procedure. (a) Laparotomy process. **(b)** The VNS device was implanted with Au leads being connected to vagal trunks. **(c)** Suture and suture removal process.

Supplementary Figure 15

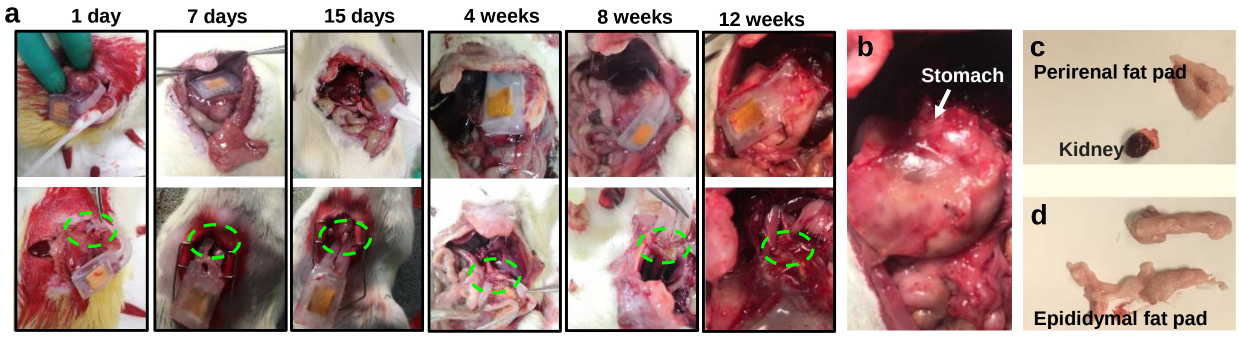

Supplementary Figure 15. Anatomical examinations and adipose tissue collection. (a) The VNS device tightly fixed to the stomach surface and Au leads were connected well with vagal trunks. SD rats were euthanized at 1 day, 7 days, 15 days, 4 weeks and 8 weeks and 12 weeks. **(b)** The regrowth tissue on stomach surface after the VNS device being removed. It further confirmed the biocompatibility of the VNS device. **(c)** The perirenal fat pad that was separated from the kidney. **(d)** Separated epididymal fat pad**.**

Supplementary Notes

Supplementary Note 1: Device fabrication details.

The PTFE thin film (50 µm) was first processed by reactive ion etching to introduce nanostructures toward the surface to enhance the electrical output (**Supplementary Figure 2a**). Specifically, 5 nm Au was sputtered on PTFE film first. Then the PTFE film was treated in an inductively coupled plasma chamber with a mixed etching gases of Ar, O_2 and CF_4 for 30s.[2] The processed PTFE was then used as a triboelectric layer for TENG fabrication. As illustrated in **Supplementary Figure 2b**, the PTFE film was paired a Cu film, and both were attached to a multi-striped template to define the device geometry. Another Cu film was attached to the back side of the PTFE film as the bottom electrode. Two Cu wires (20 mm) were connected to the top and bottom Cu electrode layers, respectively as the connecting points for electrical stimulation. An Au lead (15 mm) extension was connected to the tip of each Cu wire for vagus nerve connection. Polyimide film (50 µm) was used as the center packaging layer to open the gap between the two triboelectric layers. The device was then removed from the template surface and loaded into an oval plastic tube. Polydimethylsiloxane (15:1 PDMS; Dow Corning, USA) prepolymer was casted to cover the entire device (only the two Au leads were partially exposed) with a thickness of 1 mm, followed by curing at 60 °C for 2 hours. At last, a layer of ecoflex (200 µm) was coated onto the PDMS surface to provide extra surface flexibility to fit to the irregular surface of the stomach. The two exposed Au leads were conductive and readily to be wrapped around the vagus nerves for electrical signal stimulation.

Supplementary Note 2: Electrophysiological signals of different devices.

Electrophysiological signals are tested to reveal the response of vagus nerve in corresponding to the amplitude of electrical voltage. VNS devices with different sizes were fabricated to adjust the voltage output **(Supplementary Figure 8a)**. The voltage increased as the size increasing (**Supplementary Figure 8b)**. All these devices were implanted into different rats and their

electrophysiological single were measured and compared. As shown in **Supplementary Figure 8c,** the electrophysiological voltage signals of the intact and sham rats both had a voltage amplitude of ~0.5 mV, indicating the test is reliable. The electrophysiological voltage amplitudes increased significantly as the voltage of VNS device increased. For the VNS voltage outputs of 50 mV, 120 mV, 210 mV, 480 mV and 740 mV, the average electrophysiological voltage amplitudes were found to be 0.5 mV, 0.6 mV, 0.8 mV, 1.2 mV and 1.4 mV, respective. In addition, when the nerves were stimulated by a VNS device with >100 mV output, the number of electric pulse per signal group raised from 11 to 18, and remained at this value as the stimulation voltage further increased. When the applied voltage was 50 mV, there was only a smaller change of the number of electric pulse per signal group, and almost no change in the voltage amplitude. Thus, the activation threshold voltage might be close to 50 mV. We can conclude that the vagus nerve can be effectively stimulated by a VNS device when the voltage reach reaches ~100 mV.

Supplementary Note 3: Evaluation of body weight before and after the device removal.

Three rats with an average initial weight of 265 g (group 1, the weight-gaining groups as shown in **Fig. 4**) and three rats with an average initial weight of 495 g (group 2, the fully-grown adult groups as shown in **Fig. 5**) were fed and grown under the same conditions. Implantation surgery and extraction surgery were conducted on the 7th day and 22nd day, respectively. The results in our original manuscript have shown that rats in both group 1 and group 2 had the same body growth trend and the similar amount of food consumption before surgery (from 0-7 days).

For group 1, when the device was implanted, this group of rats exhibited the same very slow growth pattern (**Supplementary Figure 13a)** as we presented in **Fig. 4b**. After the VNS devices were removed at the 15th day post implantation, the amount of food intake quickly increased (**Supplementary Figure 13b**). The average weight of the rats exhibited an accelerated increase, matching the growth rates of the control group (shown in **Fig. 4b**). Five weeks after device removal, the average weight reached 484±28 g, which was nearly the same as the control group shown in **Fig. 4** (495±32 g).

For group 2, this group of rats exhibited an obvious body weight reduction after the device was implanted **(Supplementary Figure 13c)**, as we presented in **Fig. 5b**. After the VNS devices were removed at the 15th day post implantation, the amount of food intake quickly increased (**Supplementary Figure 13d**). The average weight of the rats exhibited an accelerated increase, matching the growth rates of control group (shown in **Fig. 5b**). Five weeks after device removal, the average weight reached 552±24 g, which was nearly the same as the control group shown in **Fig. 5** (557±39 g).

Supplementary References

1. Kuzum, D. *et al.* Transparent and flexible low noise graphene electrodes for simultaneous electrophysiology and neuroimaging. *Nat. Commun.* **5**, 5259 (2014).
2. Fang, H., Wu, W., Song, J. & Wang, Z. L. Controlled Growth of Aligned Polymer Nanowires. *J.Phys.Chem.C* **113**, 16571-16574 (2009).
