# Supplementary material for: Effective weight control via an implanted self-powered vagus nerve stimulation device
Source: Nat Commun. 2018 Dec 17;9:5349. doi: 10.1038/s41467-018-07764-z (PMC6297229; doi:10.1038/s41467-018-07764-z)
Supplement: Supplementary file 2 — Description of Additional Supplementary Files [file 41467_2018_7764_MOESM2_ESM.pdf]

Description of Additional Supplementary Files

File Name: Supplementary Movie 1
Description: Voltage output performance test on rats.

File Name: Supplementary Movie 2
Description: CT 3D view.

File Name: Supplementary Movie 3
Description: Normal life of rats with implanted VNS devices.
