# Supplementary material for: Effective weight control via an implanted self-powered vagus nerve stimulation device
Source: Nat Commun. 2018 Dec 17;9:5349. doi: 10.1038/s41467-018-07764-z (PMC6297229; doi:10.1038/s41467-018-07764-z)
Supplement: Supplementary file 6 — Reporting Summary [file 41467_2018_7764_MOESM6_ESM.pdf]

nature research

Corresponding author(s): Xudong Wang

Reporting Summary

Nature Research wishes to improve the reproducibility of the work that we publish. This form provides structure for consistency and transparency in reporting. For further information on Nature Research policies, see Authors & Referees and the Editorial Policy Checklist.

Statistical parameters

When statistical analyses are reported, confirm that the following items are present in the relevant location (e.g. figure legend, table legend, main text, or Methods section).

n/a	Confirmed	
☐	☒	The exact sample size (n) for each experimental group/condition, given as a discrete number and unit of measurement
☐	☒	An indication of whether measurements were taken from distinct samples or whether the same sample was measured repeatedly
☐	☒	The statistical test(s) used AND whether they are one- or two-sided<br>*Only common tests should be described solely by name; describe more complex techniques in the Methods section.*
☒	☐	A description of all covariates tested
☒	☐	A description of any assumptions or corrections, such as tests of normality and adjustment for multiple comparisons
☐	☒	A full description of the statistics including central tendency (e.g. means) or other basic estimates (e.g. regression coefficient) AND variation (e.g. standard deviation) or associated estimates of uncertainty (e.g. confidence intervals)
☒	☐	For null hypothesis testing, the test statistic (e.g. F, t, r) with confidence intervals, effect sizes, degrees of freedom and P value noted<br>*Give P values as exact values whenever suitable.*
☒	☐	For Bayesian analysis, information on the choice of priors and Markov chain Monte Carlo settings
☒	☐	For hierarchical and complex designs, identification of the appropriate level for tests and full reporting of outcomes
☒	☐	Estimates of effect sizes (e.g. Cohen's d, Pearson's r), indicating how they were calculated
☐	☒	Clearly defined error bars<br>*State explicitly what error bars represent (e.g. SD, SE, CI)*

Our web collection on statistics for biologists may be useful.

Software and code

Policy information about availability of computer code

Data collection	No commercial, open source nor custom code used for data collection.
Data analysis	OriginPro 9 was used for data analysis and plotting.

For manuscripts utilizing custom algorithms or software that are central to the research but not yet described in published literature, software must be made available to editors/reviewers upon request. We strongly encourage code deposition in a community repository (e.g. GitHub). See the Nature Research guidelines for submitting code & software for further information.

Data

Policy information about availability of data

All manuscripts must include a data availability statement. This statement should provide the following information, where applicable:

- Accession codes, unique identifiers, or web links for publicly available datasets
- A list of figures that have associated raw data
- A description of any restrictions on data availability

The data that support the plots within this paper and other findings of this study are available from the corresponding author upon reasonable request.

Field-specific reporting

Please select the best fit for your research. If you are not sure, read the appropriate sections before making your selection.

☒ Life sciences ☐ Behavioural & social sciences ☐ Ecological, evolutionary & environmental sciences

For a reference copy of the document with all sections, see nature.com/authors/policies/ReportingSummary-flat.pdf

Life sciences study design

All studies must disclose on these points even when the disclosure is negative.

Sample size	Normalized cell viability on encapsulation material and culture dish (n=3 for each group) were analyzed (Fig. 1g). Whole blood and chemical analysis were performed on the four groups (VNS, Sham, Lap and Intact, n=3 for each group) of rats for biosafety assessment during the implantation period (Figs. 3d-i and Supplementary Figure 11). The weight control performance was first examined in the four groups of rats(VNS, Sham, Lap and Intact, n=6 for each group) (the average initial weight of rats was 250 g) that were fed and grown under the same conditions (Figs. 4b, c, d, e, g and h). The weight loss performance was then examined in the four groups of rats(VNS, Sham, Lap and Intact, n=4 for each group) (the average initial weight of rats was 500 g) that were fed and grown under the same conditions (Figs. 5b, c, d, e, g and h). Evaluation of body weight before and after the device removal in Supplementary Figure 13. (n=3 for each group)
Data exclusions	No data was excluded.
Replication	The sample size both in vitro and in vivo experimental groups were ≥ 3 to ensure replicability. From the measured results, the data showed high similarity within the same testing group, and the replicability was good in each group.
Randomization	All devices and animals tested were selected randomly.
Blinding	N/A (only one condition that the devices work properly can affect the result (VNS group), and there was no significant differences in other three control groups (Sham, Lap and Intact). The blinding process won't influence result.

Reporting for specific materials, systems and methods

Materials & experimental systems

n/a	Involved in the study
☒	☐ Unique biological materials
☒	☐ Antibodies
☒	☐ Eukaryotic cell lines
☒	☐ Palaeontology
☐	☒ Animals and other organisms
☒	☐ Human research participants

Methods

n/a	Involved in the study
☒	☐ ChIP-seq
☒	☐ Flow cytometry
☒	☐ MRI-based neuroimaging

Animals and other organisms

Policy information about studies involving animals; ARRIVE guidelines recommended for reporting animal research

Laboratory animals	7–8 weeks old male Sprague-Dawley rats were acquired from Envigo (New Jersey, USA).
Wild animals	This study did not involve wild animals.
Field-collected samples	This study did not involve samples collected from the field.
